# USDA Snack Policy Implementation: Best Practices From the Front Lines, United States, 2013–2014

**DOI:** 10.5888/pcd13.160023

**Published:** 2016-06-16

**Authors:** Yuka Asada, Jamie Chriqui, Noel Chavez, Angela Odoms-Young, Arden Handler

**Affiliations:** Author Affiliations: Jamie F. Chriqui, Institute for Health Research and Policy, School of Public Health, University of Illinois at Chicago, Chicago, Illinois; Noel Chavez, Community Health Sciences, School of Public Health, University of Illinois at Chicago, Chicago, Illinois; Angela Odoms-Young, College of Applied Health Sciences, Department of Kinesiology and Nutrition, College of Allied Health, University of Illinois at Chicago, Chicago, Illinois; Arden Handler, Community Health Sciences, School of Public Health, University of Illinois at Chicago, Chicago, Illinois.

## Abstract

**Introduction:**

The Smart Snacks in Schools interim final rule was promulgated by the US Department of Agriculture (USDA) as authorized by the Healthy, Hunger-Free Kids Act of 2010 (PL 111–296) and implementation commenced beginning July 1, 2014; however, in the years leading up to this deadline, national studies suggested that most schools were far from meeting the USDA standards. Evidence to guide successful implementation of the standards is needed. This study examined snack policy implementation in exemplary high schools to learn best practices for implementation.

**Methods:**

Guided by a multiple case study approach, school professionals (n = 37) from 9 high schools across 8 states were recruited to be interviewed about perceptions of school snack implementation; schools were selected using criterion sampling on the basis of the HealthierUS Schools Challenge: Smarter Lunchrooms (HUSSC: SL) database. Interview transcripts and internal documents were organized and coded in ATLAS.Ti v7; 2 researchers coded and analyzed data using a constant comparative analysis method to identify best practice themes.

**Results:**

Best practices for snack policy implementation included incorporating the HUSSC: SL award’s comprehensive wellness approach; leveraging state laws or district policies to reinforce snack reform initiatives; creating strong internal and external partnerships; and crafting positive and strategic communications.

**Conclusion:**

Implementation of snack policies requires evidence of successful experiences from those on the front lines. As federal, state, and local technical assistance entities work to ensure implementation of the Smart Snacks standards, these best practices provide strategies to facilitate the process.

## Introduction

With the passage of the Healthy, Hunger-Free Kids Act of 2010 (P.L. 111–296), Congress authorized the US Department of Agriculture (USDA) to establish nutrition standards for nonmeal-program items sold in schools during the school day (also known as “competitive foods and beverages”) (1). In June 2013, the USDA issued an interim final rule, “Smart Snacks in Schools,” which was to be implemented as of July 1, 2014 (2). However, during the school year leading up to the deadline, unhealthy snack items remained ubiquitous, especially in high schools. In the 2013–2014 school year, 87% of high school students nationwide still had access to sugar-sweetened beverages through snack venues (3), thus causing concern that full implementation of the standards may be delayed.

A systematic review reported a small but positive link between healthful snack policies and outcomes such as decreased student consumption of unhealthful snacks and decreased availability of unhealthful snacks (4). Few studies examine how the regulated standards are translated from policy to school practices (5,6). Such evidence could be used by schools struggling with translating Smart Snacks standards into practice. The objective of this study was to learn best practices from high schools that successfully implemented stringent snack guidelines so those practices could be used by other schools before they began implementing the Smart Snacks standards.

## Methods

A multiple case-study approach allowed us to examine high schools in their natural setting (7). This approach aligned well with the purpose of this study because federal and state mandates do not allow for randomization of high schools. The main research question was: among high schools with strong snack practices, what are the critical factors that allowed the practices to be implemented successfully? By identifying these factors, we were able to determine best practices for snack policy implementation.

High schools were selected as cases because they typically have the worst snack environments of all school levels (3). To select from schools already recognized for rigorous snack practices, we used the HealthierUS School Challenge: Smarter Lunchrooms database (HUSSC: SL) as a sampling frame. In February 2014, 203 high schools achieved some level of HUSSC: SL award status. From this sampling frame, we used criterion sampling principles (8) to select 9 high schools with a range of state, district, and school characteristics from the Bridging the Gap state law database (school year 2012–2013) (9), state child obesity rates from the National Survey of Children’s Health 2011–2012 (10), and district and school-level socioeconomic data from the National Center for Education Statistics (NCES) Common Core Data (CCD) (school year 2011–2012) (11).

The University of Illinois at Chicago (UIC) Institutional Review Board approved this study (institutional review board protocol no. 2013–1007). The lead author first obtained permission from school district superintendents before contacting school professionals via email to invite them for key informant, semistructured interviews from November 2013 to October 2014. School professionals were contacted if they were involved with snack policy implementation as identified by the food service director (FSD), school principal, or superintendent. The interview guide was based on research findings already published, pilot-tested with an FSD, and iteratively revised on the basis of feedback from external school professionals and USDA officials. On the basis of the FSD guide, we modified guides intended to be used with other school professionals. The interview guide contained questions about implementing the snack policy, the key stakeholders in having the policy implemented or not implemented, the facilitators and barriers to implementing the policy, and sources of technical assistance. The guides are available by request to the first author. Telephone interviews lasted from 45 to 70 minutes and were audiotaped and transcribed. Post-interview notes and a detailed, ongoing study audit trail were updated and consulted during analysis. To triangulate interview findings, we collected internal documents such as each school district’s wellness and snack policies, newsletters, and Facebook pages.

Over 140 documents comprising interview transcripts, internal documents, and post interview summaries were uploaded into Atlas.ti v.7 (Scientific Software Development GmbH) for organization and coding. External school professionals, USDA officials, and expert researchers informed iterative revisions of an a priori coding guide. The coding process was guided by Saldaña’s first- and second-pass coding, with the goal of turning the large data set into organized, categorized, and small, manageable constructs for analysis (12). Two analysts read through and independently coded 20% of the transcripts; rounds of coding continued until 80% inter-rater agreement was reached (13). The analysts met regularly to discuss revisions to the coding guide, emergent themes, and explanations of memos. These ongoing discussions facilitated the iterative process of theme generation and refinement. Each analyst then created detailed case summaries that outlined the timelines and implementation process for each high school; this process allowed for an in-depth understanding of the contexts and nuances of the phenomenon in each unique case. Cross-case analysis (7) guided a process that looked for emergent themes and commonalities across the cases. One important component of this process included negative case analysis, which compared snack policy implementation across cases in order to spot outliers and deepen theme generation. The coding and analysis process is described in full elsewhere, including the steps taken to ensure rigorous team analysis (14). Lastly, adhering to principles of trustworthiness, we enhanced the rigor of study findings by including continuous peer debriefing and a detailed audit trail (15).

## Results

The characteristics of the 9 high schools in the study are in Table 1. As the table indicates, the schools were in 9 districts and 8 states and spanned all 4 Census regions. Based on the Bridging the Gap state law database 2012–2013 (9), some schools were in states with strong laws and some in states with weak state laws. State snack laws — specifically those applicable to snack food sales in vending machines, à la carte cafeteria lines, school stores, classroom parties, and fundraising — were coded using a rigorous coding scheme adapted from Schwartz et al (16). For each location of sale, state laws were coded for a series of nutrient and beverage standards. Provisions in state law that were “encouraged” we classified as weak. Also weak were laws that used terms such as “should” or “may,” or that allowed exceptions, or that applied only at certain times of the day or at certain sale locations. In contrast, strong laws definitively required specific food and beverage standards and used terms such as “shall,” “must,” and “require.” Fourteen individual variables were coded for strength for each sale location. A composite scale measured the proportion of the 14 individual items coded for each sale location that were definitively “strong.” The resulting strength scale ranged from 0 to 100, with 100 being the highest score for provisions that were required by law (9). It is notable that the highest score was 47 of 100 (Mississippi) with all others scoring below 34 of 100. In addition, some schools were located in large districts and some in small (range: 3–642 schools and 57–2,531 students per high school). The schools also varied by locale (city, town, suburban, rural), by racial/ethnic makeup of the student population, by socioeconomic status (range: 17% free- or reduced-price lunch participation [FRLP] to 89% FRPL), and by state rate for childhood obesity (range: 28% in Iowa to 40% in Mississippi).

**Table 1:** Characteristics of 9 Schools With Best Practices for Snack Policies, United States, 2013–2014

Schools	State^a^	Region	State Policy Strength Score^b^	State Childhood Obesity, %^c^	No. of Schools in District	Locale^d^	Total Students	FRPL (%)^e^
1	California 1	West	33	30	29	City	1,200	55
2	California 2	West	33	30	12	Suburb	2,531	72
3	Illinois	Midwest	12	34	642	City	1,042	89
4	Iowa	Midwest	28	28	7	Town	431	18
5	Kansas	Southwest	1	30	9	Rural	57	84
6	Mississippi	South	47	40	10	City	895	82
7	New York	East	1	32	3	Town	513	17
8	Texas	West South Central	34	37	65	Rural	1,890	80
9	Virginia	Southeast	14	30	18	Suburb	1,294	22

Abbreviations: FRPL, free and reduced-price lunch; FSD, food service director; PE, physical education; admin, administrator.

a Two schools were in California.

b State law strength score calculation per Bridging the Gap school year 2012–2013 data (high school data) (9). State law strength is calculated by a rigorous coding process originally published by Schwartz et al (16) and adapted by researchers at Bridging the Gap (2010). The score measures the proportion of snack food items — specifically those in vending machines, à la carte, and in school stores — that were required by law. A requirement is considered and coded to include policy language that used words such as “shall,” “must,” “required,” as compared with weaker language such as “encourage,” “should,” “may.” Scores range from 0–100 with 100 being the highest score.

c National Survey of Children’s Health (2011–2012) (10).

d National Center for Education Statistics (NCES), 2011–2012 (11).

e Percentage of students eligible for free- or reduced-price lunches.

Given the focus on FSDs and principals in school wellness literature, we made a concerted effort to include a wide range of school professionals in this study (Table 2). Respondents (n = 39) were 11 district FSDs, 8 athletic directors/PE teachers/boosters, 7 principals/vice principals, 5 cafeteria managers, and the remaining 8 respondents were a nurse, consumer science/health teacher, technical assistance provider, or a finance administrator. We interviewed a minimum of 3 school professionals per case, with 4 or more school professionals interviewed in 7 of the 9 cases.

**Table 2:** Respondent Types at 9 High Schools With Best Practices for Snack Policies, United States, 2013–2014

State^a^	FSD	Principal/Vice Principal	Cafeteria Manager	Athletics Directors; PE Teachers	Nurse/ Health Service	Family Consumer Science Health Teacher	Technical Assistance Provider	Finance Admin	Total
**California 1**	1	1	1	2	1	—	—	—	6
**California 2**	1	1	—	1	—	—	1	—	4
**Illinois**	1	1	1	1	—	1	—	—	5
**Iowa**	1	—	1	1	—	—	2	—	5
**Kansas**	2	1	—	—	1	—	—	—	4
**Mississippi**	1	1	—	1	—	1	—	—	4
**New York**	1	1	1	—	—	—	—	—	3
**Texas**	1	1	1	1	—	—	—	1	5
**Virginia**	2	—	—	1	—	—	—	—	3
**Column Totals**	11	7	5	8	2	2	3	1	39

Abbreviation: FSD, food service director; PE, physical education.

a Two schools were in California.

The findings are derived from cross-case analysis across high schools and respondent types. Because FSDs had the most pivotal role in implementation, the findings are reported from the FSDs’ perspective, with supporting quotes from additional respondents. FSDs used a variety of models for implementing a successful snack policy, which is evident from several overarching themes described in the Box.

**Box. Best Practices for Snack Policy Implementation: Key Themes, United States, 2013–2014**

**Table:** 

Theme 1	Implementation and acceptance takes time and continued effort.
Theme 2	HUSSC: SL certification was a critical catalyst.
Theme 3	FSDs leaned on the power of state law and district policies.
Theme 4	Internal and external partnerships increased capacity.
Theme 5	Strategic communications changed perceptions.

Abbreviations: FSD, food service director; HUSSC: SL, HealthierUS Schools Challenge: Smarter Lunchrooms.

### Theme 1: Implementation and acceptance take time and continued effort

All FSDs shared that implementation required a series of activities that took time and continued effort to keep “plugging away at it.” Many FSDs began reforming the school food and snack environment several years before the Smart Snacks deadline, and despite attaining HUSSC: SL status, most were still planning initiatives toward boosting student acceptance of various components. A booster club leader from Mississippi shared:

We’ve never been asked to deviate from our menu because of it being in concessions . . . . If we had to adhere to those standards, I don’t think we’d be as successful as we are now. I think over time we would because it would be an initial culture shock if we had to remove all carbonated drinks. I think we will initially have a change, but overall, and I guess a few years down the road, I think it would become just common and end up being OK.

### Theme 2: HUSSC: SL certification was a critical catalyst

The HUSSC: SL award was reported to be a catalyst for snack policy implementation in 2 main ways. First, attaining any award status required that the school demonstrate to the USDA that they are implementing both the snack and district wellness policy comprehensively; these combined efforts facilitated an enhanced awareness of health and wellness. In particular, school professionals described nutrition education programs as a strong facilitator of snack reforms. An FSD from Mississippi shared that nutrition education created “another partner in the classroom.” Second, many high schools faced challenges during school meal reform [ie, implementation of the USDA standards: Nutrition Standards in the National School Lunch and Breakfast Programs (17)], which was due for implementation 2 years before Smart Snacks. The HUSSC: SL award brought positive recognition and boosted the reputation of any food service department that attained it. An FSD from Iowa said “It helped to get the HealthierUS School Challenge, it helped to get patted on the back, to make these kids realize, the teachers, and the parents, realize that the kids weren’t getting tortured” [laughs]. Such recognition from the school community at large facilitated the FSDs’ ongoing initiatives to improve the school food environment.

### Theme 3: FSDs leaned on the power of state law and district policies

Although a change in policy alone may be insufficient to change school practice (18), the existence of a policy can act as a strong facilitator for implementation. FSDs across cases had varying levels of power and influence in their school districts; however, all leaned on a district policy, state law, or both to “reinforce” their initiatives. As a Texas FSD said, “Schools make decisions based on policy. The curriculum is based on policy. Even salaries are based on school board policies. So as long as there’s a policy that is within hands’ reach, so you can actually print it out and put it on paper, it helps to reinforce what we’re doing.”

In addition, school professionals from states with strong laws overwhelmingly referenced the law as a catalyst for implementation. The power of state law was evident in discussions with principals, who despite having minimal interest in snack reform and wellness activities, referenced it as a reason for compliance. According to a principal from Mississippi, “And it’s that you just have to be in compliance with the state nutrition guidelines. That’s when you’re serving food in the cafeteria as well as in the vending machines, here on campus. Either one, the state has adopted such policies. I mean, we were pretty much forced to. It’s state law.” Essentially, the power of the district policy or state law was evident in comments from all school professionals.

### Theme 4: Internal and external partnerships and collaborations increased capacity

It is important to acknowledge that these initiatives were added to FSDs’ existing duties and responsibilities. As a result, FSDs emphasized that internal and external partnerships and collaborations increased their own capacity for implementation. One FSD from California recalled his reliance on partnerships and collaborations:

I really reached out to the larger nutrition community as a whole. For example, I’m just gonna name a few of our collaborators: an insurance group, not-for-profit community health, let’s see — Alliance for Healthier Generation, the farmers market, the State Department of Education. I incorporated a lot of these collaborations and partnerships to utilize their resources. Because I myself alone, there’s no way that I could’ve made these changes.

The most salient internal partnership cited by all respondents was with school leadership; as the “go-to person” and “hub of communications,” the principal was a critical stakeholder. Further, the most notable external partnerships across cases were with state departments of education and respective child nutrition divisions, which provided invaluable hands-on technical assistance and resources, as well as peer networks with partner school districts. An FSD from Illinois explained, “I have kind of a monthly call with them [peer FSDs] and we share some of our challenges and best practices. We visit each other’s schools and districts to see what you’re doing, what I’m doing, how can I incorporate some of what you’re doing, how can you incorporate what I’m doing.”

FSDs creatively relied on a range of partnerships and collaborations for technical assistance and additional resources; this best practice was especially critical given that these individuals led snack (and wellness) policy implementation in addition to their existing job responsibilities.

### Theme 5: Strategic communications changed perceptions

School professionals expressed that removing unhealthful junk foods (ie, low nutrient and high sugar, fat, calorie, and sodium food and beverage items) was “the right thing to do.” A critical practice during implementation was extending their perceptions to others within and outside the school community about the value and intentions of the snack policy. One FSD in Iowa said, “It’s terrible, they didn’t want the government telling them what to do. And I can understand that, I don’t really like the government telling me what to do, but in my view, it wasn’t that, it’s showing these kids healthy guidelines.”

In this way, the FSDs’ communications with key stakeholders about the potential impact of the snack policy fostered support for their initiatives. As an FSD in Virginia recalled from school meal standards implementation, many stakeholders were misinformed about reform efforts. Thus, learning from that experience, she took an active approach when dealing with snack policy implementation by focusing heavily on messaging and communications that framed school snacks reform in a positive light: “share, share, and share your successes!”

## Discussion

This study contributes to the limited best practices evidence for Smart Snacks implementation in US high schools. Overall, school professionals reported that implementation is taking time and is an ongoing effort, which is an encouraging finding for struggling schools still in the process of fully adhering to Smart Snacks. HUSSC: SL certification was reported to be a strong catalyst because of its emphasis on a comprehensive wellness approach; this finding is consistent with a study by Bassler and colleagues (19), who also examined snack reform in middle and high school districts. In the current study, FSDs noted that HUSSC: SL acted as a facilitator, bringing positive recognition for the child nutrition department. FSDs operate in complex contexts in the school community, and initiatives that boost the reputation and awareness of FSDs can act as facilitators for their initiatives. Because working toward receiving the HUSSC: SL was reported as a best practice, organizations providing technical assistance to schools should encourage schools to strive for this award.

Implementers also leaned on state law and district policy as sources of power to legitimize and reinforce their initiatives. This finding is a more nuanced explanation of what is known from current research findings: showing a link between a stringent state law and more healthful school practices and student-level outcomes (20–25). Given that school districts tend to follow state standards, these findings indicate that leadership from the state level has strong potential for helping FSDs implement their healthful initiatives. Local and state technical assistance providers should consider training FSDs to maximize their ability to leverage local policies and state laws for the benefit of programs that reduce unhealthful snacks in schools. This finding also provides a rationale for advocacy at all levels in support of state laws that improve snack policies for schools.

FSDs also pointed to the challenge of leading snack policy implementation in addition to their regular duties; their efforts to build strong partnerships and collaborations were essential to boost their capacity and increase access to needed resources. This finding may be especially salient to FSDs in school districts with limited resources for wellness and nutrition activities. External organizations, such as local public health departments and nonprofit technical assistance providers may be open to partnerships and collaborations with schools for wellness-related efforts.

Lastly, strategic and positive communication also emerged as a best practice, highlighting the essential role that FSDs play in shaping and framing food-related policy implementation for the school community. Recently, the USDA issued a final rule addressing professional standards for school nutrition programs; under this rule, one of the 4 training categories listed includes communications and marketing (26). Technical assistance providers should consider emphasizing this category for professional development with FSDs as well as with other school professionals who are involved with Smart Snacks implementation.

The best practices that emerged here were drawn from exemplary high schools across the United States; they may not reflect the experiences of more typical high schools nationwide. Because the objective of this study was to provide best practices to inform implementation for other high schools, the sampling strategy focused on the exemplary case; however, future studies should focus on a typical case for a more comprehensive understanding of implementation in schools. In addition, the study is missing input from a few key stakeholder groups, including students, parents, and state technical assistance providers. Future studies should focus on perspectives provided by these additional groups. Furthermore, this article does not present findings on monitoring and enforcement activities that occurred during and after implementation processes, which are critical to the success of full implementation.

Despite these limitations, this study is one of the few that examined US snack policy implementation in high schools from a process rather than outcomes perspective. The study design and execution were guided by external school practitioners and USDA officials to ensure that findings were relevant to both practice and policy. Finally, to engage a wide range of voices (eg, FSDs, PE teachers, cafeteria managers) this study expanded the range of school professionals typically involved in published studies; this process purposefully included the stakeholders that must be involved for successful implementation of Smart Snacks in schools.

As schools nationwide work toward full implementation of Smart Snacks, this study provides evidence that schools still working toward full adherence should adopt our list of best practices.
